# Glutaric aciduria type 1: Diagnosis, clinical features and long‐term outcome in a large cohort of 34 Irish patients

**DOI:** 10.1002/jmd2.12302

**Published:** 2022-06-14

**Authors:** Lydia Healy, Meabh O'Shea, Jennifer McNulty, Graham King, Eilish Twomey, Eileen Treacy, Ellen Crushell, Joanne Hughes, Ina Knerr, Ahmad Ardeshir Monavari

**Affiliations:** ^1^ National Centre of Inherited Metabolic Disorders, Children's Health Ireland at Temple Street Dublin Republic of Ireland; ^2^ European Reference Network for Rare Hereditary Metabolic Disorders (MetabERN) European Reference Network for Rare Hereditary Metabolic Disorders (MetabERN), National Centre for Inherited Metabolic Disorders, Children's Health Ireland at Temple Street and Mater Misericordiae University Hospital Dublin Republic of Ireland; ^3^ Department of Radiology Children's Health Ireland at Temple Street Dublin Republic of Ireland; ^4^ National Adult Centre for Inherited Metabolic Disorders Mater Misericordiae University Hospital Dublin Republic of Ireland; ^5^ University College Dublin Dublin Republic of Ireland; ^6^ University of Dublin Dublin Republic of Ireland

**Keywords:** gluatric aciduria type 1, high‐excretor, high‐risk screen, low‐excretor, newborn screening, retrospective analysis

## Abstract

Glutaric aciduria type 1 (GA1) is a rare neurometabolic disorder that can lead to encephalopathic crises and severe dystonic movement disorders. Adherence to strict dietary restriction, in particular a diet low in lysine, carnitine supplementation and emergency treatment in pre‐symptomatic patients diagnosed by high‐risk screen (HRS) or newborn screen (NBS) leads to a favourable outcome. We present biochemical and clinical characteristics and long‐term outcome data of 34 Irish patients with GA1 aged 1–40 years. Sixteen patients were diagnosed clinically, and 17 patients by HRS, prior to introduction of NBS for GA1 in the Republic of Ireland in 2018. One patient was diagnosed by NBS. Clinical diagnosis was at a median of 1 year (range 1 month to 8 years) and by HRS was at a median of 4 days (range 3 days to 11 years). 14/18 (77.8%) diagnosed by HRS or NBS had neither clinical manifestations nor radiological features of GA1, or had radiological features only, compared to 0/16 (0%) diagnosed clinically (*p* < 0.001). Patients diagnosed clinically who survived to school‐age were more likely to have significant cerebral palsy and dystonia (7/11; 63.6% vs. 0/13; 0%, *p* < 0.001). They were less likely to be in mainstream school versus the HRS group (5/10; 50% vs. 12/13; 92.3%; *p* = 0.012). Clinical events occurring after 6 years of age were unusual, but included spastic diplegia, thalamic haemorrhage, Chiari malformation, pituitary hormone deficiency and epilepsy. The exact aetiology of these events is unclear.

**Synopsis**

We report the outcomes of 34 Irish patients with GA1, including new symptom onset in five patients over six years on established treatment, supporting the recommendation for diet, carnitine supplementation as well as emergency treatment ‘for life’.

## 1 INTRODUCTION

Glutaric aciduria type 1 (GA1, OMIM#231670) is a rare  neurometabolic disorder of lysine, hydroxylysine and tryptophan metabolism caused by profound deficiency of the mitochondrial enzyme, glutaryl‐CoA dehydrogenase (GCDH; EC number: 1.3.99.7). GCDH deficiency results in a build‐up of glutaryl‐coenzyme A (CoA), and its derivatives: glutaric acid (GA), glutaconic acid, 3‐hydroxyglutaric acid (3‐OH‐GA) and glutarylcarnitine (C5DC).

GA1, an autosomal recessive disorder, is caused by pathogenic variants in the *GCDH* gene, located on chromosome 19p13. Over 200 variants associated with GA1 have been identified (Human Gene Mutation Database accessed January 2022).^1^ Despite a high degree of heterogeneity in disease‐causing variants, single common variants have been discovered in genetically homogenous communities. Naughten et al.^2^ reported that homozygosity for the common c.1093G>A, variant was the most common genotype among members of the Irish Traveller Community (ITC), an ethnic minority group.

The incidence of GA1 in Ireland is estimated at 1 in 56 000,^2^, ^3^ higher than the reported worldwide incidence of 1 in 110 000.^4^ This higher Irish figure is due to increased estimated incidence of 1 in 2000 in the ITC. Other minority ethnic groups such as Amish community and Lumbee tribe in the United States, Ojii‐Cree First Nations People in Canada and Xhosa group in South Africa also experience increased incidence of GA1.^5^ The introduction of newborn bloodspot screening (NBS) has been a crucial tool in the diagnosis of GA1 internationally, with many patients worldwide being diagnosed in the first weeks of life. Currently 24 countries in Europe screen for GA1 and screening is piloted in two further countries.^6^ Since December 2018, GA1 is included in the Irish national NBS Programme. Internationally, NBS has been shown to greatly reduce incidence of complex motor disorder (MD), although it is dependent on adherence to treatment.^4^, ^7^, ^8^

Patients with GA1 typically present within the first 3 years of life with acute encephalopathic crisis precipitated by catabolism.^9^, ^10^ This initial crisis, or subsequent crises, can cause striatal degeneration. This striatal injury often results in MD with predominantly dystonic/dyskinetic features.^9^ Initially it was thought that intellect is unaffected in GA1,^10^ but recent studies have shown that cognitive impairment is a feature, and that it is predicted by high excretor status.^11^ Due to the early presence of subdural effusion, GA1 has been previously misdiagnosed in children as non‐accidental injury.^12^

Patients with GA1 are characterised as high excretors (HE) or low excretors (LE) based on urinary glutaric acid levels.^13^ Traditionally it was thought that all biochemical phenotypes had a similar clinical course but recent evidence has emerged that HE patients are at increased risk of progressive white matter damage in later life, although the clinical significance of this is unknown.^11^, ^14^

*Insidious onset* GA1 describes striatal injury and MD occurring without apparent crisis and is estimated to account for 20% of symptomatic presentations.^9^, ^15^ This phenotype has also been described in patients diagnosed by newborn screening (NBS) with suboptimal adherence to dietary regimes, in whom it may account for up to 50% of cases of MD.^7^, ^8^, ^16^ *Late‐onset* GA1 refers to diagnosis any time after 6 years of life.^9^, ^17^ Reported patients are exclusively HE and symptoms may be non‐specific, including headache, memory loss, dysarthria, weakness, epilepsy and difficulty with co‐ordination. Asymptomatic mothers diagnosed as a result of NBS are also reported, with white matter changes predominating on MRI.^18^, ^19^, ^20^, ^21^, ^22^, ^23^, ^24^

We present outcome data on 34 Irish patients with GA1 treated at a single National Centre over 37 years, diagnosed clinically and by high‐risk screen (HRS) due to positive family history. We report characteristics of patients at diagnosis and at school‐age (5–7 years), this age being the traditional ceiling at which acute complications were thought to occur in GA1. Subsequent to the introduction of NBS in Ireland, one patient has been diagnosed in this manner, with their older sibling subsequently diagnosed by HRS.

Among this cohort of patients attending our National Centre, distinctive clinical events occurring after 6 years of age were also observed, details of which are supplied, although the exact aetiology of these phenomena is uncertain. Some of the symptoms described have not previously been reported among patients with GA1, while others are similar to known phenotypes of *late‐onset* GA1.

## 2 METHODS

Retrospective chart review was undertaken of patients attending the National Centre for Inherited Metabolic Disorders 1983–2020. Anonymised data were collected from patients' diagnosis and at school age (5–7 years), as well as overall outcome (deceased or current age), with note of any remarkable complications. Where a data point could not be determined for a patient, the patient was excluded from analysis relative to that data point only. This study was approved by the Research and Ethics Committee (REC) of Children's Health Ireland at Temple Street. Written consent was obtained from all patients and their parents/guardians as appropriate.

### 2.1 Definitions

A patient was described as having clinical features of GA1 at diagnosis if any of the following were present: macrocephaly (head circumference above 98th centile for age), dystonia, gross motor delay (both as defined by clinician), acute metabolic or encephalopathic crisis or specific neuro‐radiological features. Acute encephalopathic crisis was defined as acute onset of neurologic disease (e.g., complex motor disorder) after an episode that was likely to precipitate a catabolic state (e.g., febrile illness) during infancy or childhood in the absence of any alternative cause (e.g., meningitis). Patients were described to have insidious onset dystonia when dystonia was present at diagnosis without any apparent crisis. Patients were described as having epilepsy when they had an ongoing tendency for unprovoked seizures.

Low‐excretors were described as having <100 mmol GA/mol creatinine in urine at diagnosis. Radiological features of GA1 were adapted from Twomey et al.^25^ and defined as present if *any* of the following were described by a reporting consultant radiologist: increased interhemispheric or extra‐axial space; widened sylvian fissure, atrophy or hypoplasia of the temporal lobe; abnormalities of the basal ganglia; subdural effusion and bilateral caudothalamic cysts. Due to variation in imaging modality performed at diagnosis, reported abnormalities refer to whatever modality was used. Diagnosis was confirmed biochemically by analysis of urinary 3‐hydroxy‐glutarate and/or plasma glutarylcarnitine (C5DC), enzymatically in cultured fibroblasts, and more recently by genetic variant (*GCDH* gene) when available.

### 2.2 Patient management and protocols

Over time, our model of care has developed from long admissions to earlier discharge and day care as guidelines and protocols for home‐care management were developed by health care professionals, with input of the patients and families. Care‐plans are shared with the local hospitals. Maintenance treatment is agedependent and consists of lysine‐free/low‐tryptophan/arginine‐enriched amino acids mixture 1–2 g/kg/day, natural protein 0.8–1.5 g/kg/day, with average lysine 65–90 mg/kg/day, tryptophan 12‐19 mg/kg/day and calories 110–120 kcal/kg/day (<2 years); 75–110 kcal/kg/day (2‐12 years). During emergency treatment, natural protein is reduced/stopped for maximum of 48 h when unwell and calories increased to 120% until recovery. Natural protein allowance is increased after 10 years and carnitine supplementation is continued lifelong with dosage tailored depending on age and/or free carnitine concentration in plasma or dried bloodspot samples; synthetic amino acid supplement is maintained and tailored to the patient's dietetic needs. Quantitative amino acids are measured three monthly less than 6 years and six monthly over 6 years. Neuroimaging is performed at diagnosis, and as the need arises. This has been the treatment protocol in our centre for 28 years with some adjustments, and all patients received this treatment unless otherwise specified.

### 2.3 Statistics

Data were analysed using *SPSS Version 26* (IBM Corp., Armonk, NY). Categorical data were analysed using Pearson‐chi squared. Numerical data were graphed and assessed for normality of distribution using the Shapiro–Wilk test and analysed using Mann–Whitney *U*‐test or Student's *t*‐test accordingly.

## 3 RESULTS

### 3.1 Study population

There were 34 patients with GA1 under the regular care of the metabolic centre during the study period, diagnosed between 1983 and 2020 and equating to 438 patient‐years. Sixteen patients were diagnosed after presenting clinically, 17 were diagnosed by HRS and one by NBS. Diagnosis by HRS was at a median of 4 days, (range 3 days to 11 years) and by clinical presentation at a median of 1 year (range 1 month to 8 years; Table 1). Three patients had a LE phenotype (see outcomes below). Thirty‐one patients received the treatment protocol. Two of these patients were symptomatic and diagnosed prior to establishment of this protocol, and one declined to follow treatment due to carer preference. The genetic variant was known for 27 of 34 (79.4%) of patients (Table 1). The list of pathogenic variants in the *GCDH* gene present in the patient population are shown in Table 2, the most prevalent allele being the c.1093G>A variant, accounting for 63% of known alleles.

**TABLE 1:** Characteristics of patients with GA1 at diagnosis and outcomes of patients with GA1

	Clinical diagnosis (*n* = 16)	High‐risk screen (*n* = 18)^a^	*p* Value
Age at diagnosis (days; median and interquartile range)	407 (203–668)	4 (3–40)	<0.001
Member or Irish Travelling Community	8/16 (50%)	12/18 (66.7%)	n.s.
Male	10/16 (62.5%)	7/18 (37.5%)	n.s.
Low‐excretors	2/16 (12.5%)	1/18 (5.6%)	n.s.
Genotype known	12/16 (75%)	15/18 (83.3%)	n.s.
Presented with metabolic crisis^b^	9/16^b^ (56.3%)	n/a	
Presented with isolated motor delay	4/16 (25%)	n/a	
Presented with insidious onset dystonia	3/16 (18.8%)	n/a	
Alive	8/15^c^ (53.3%)	16/18 (88.9%)	0.022
Age deceased (mean, range)	5.45 years (0.75–13)	2.38 years (0.02–4.75)	n.s.
Cerebral Palsy equivalent GMFCS 2 and higher (5–7 years)	7/11 (63.6%)	0/13^d^ (0%)	0.001
Dystonia (5–7 years)^e^	7/11 (63.6%)	0/13 (0%)	0.001
Epilepsy (any age)	5/16 (31.3%)	3/18 (16.7%)	n.s
In mainstream school (5–7 years)	5/11 (45.5%)	12/13° (92.3%)	0.012

^a^ Includes data on one patient diagnosed by newborn screen.

^b^ One patient presented with metabolic acidosis but did not develop dystonia or acute encephalopathy.

^c^ One patient lost to follow‐up.

^d^ Patients not yet school‐age.

^e^ Same patients affected by dystonia and cerebral palsy.

**TABLE 2:** Frequency of pathogenic variants in the *GCDH* gene encoding glutaryl‐CoA dehydrogenase present in the Irish cohort

DNA sequence change allele 1	Amino acid change	DNA sequence change allele 2	Amino acid change	Frequency in cohort
c.1093G>A	Glu365Lys	c.1093G>A	Glu365Lys	17/27 (63%)
c.IVS2+1G>A	–	c.IVS2+1G>A	–	2/27 (7%)
c.1204C>T	Arg402Trp	c.1204C>T	Arg402Trp	1/27 (4%)
c.172G>T	Glu58Ter	c.641C>T	Thr214Met	1/27 (4%)
c.680G>C	Arg227Pro	c.680G>C	Arg227Pro	1/27 (4%)
c.344G>A	Cys115Tyr	c.743C>T	Pro248Leu	3/27 (9%)
c.344G>A	Cys115Tyr	c.1204C>T	Arg402Trp	1/27 (4%)
c.281G>A	Arg94Gln	c.1082+2T>G	–	1 (4%)

### 3.2 Clinical features at diagnosis

The frequency of features of GA1 in the patient population at diagnosis is illustrated in Table 3. Seven of 18 (38.9%) patients diagnosed by NBS and HRS had no features of GA1 at diagnosis, while an additional seven had only radiological features of GA1 at diagnosis. The four remaining patients diagnosed by NBS and HRS had macrocephaly, and one had motor delay. Regarding the 16 patients diagnosed clinically, eight (50%) presented with an acute metabolic crisis with encephalopathy, and an additional patient had metabolic crisis (vomiting, hypoglycaemia and metabolic acidosis) but did not become encephalopathic (Tables 1 and 3). The seven other patients presented with motor delay or insidious onset dystonia (Table 1).

**TABLE 3:** Documented features of GA1 at diagnosis in A: patients who presented clinically and B: patients diagnosed by HRS (and one by NBS).

Cohort A								Cohort B							
	N	MC	DA	DI	MD	AI	MH		N	MC	DA	DI	MD	AI	MH
1		*	*			*	*	1						*	
2		*	*			*		2						*	
3		*	*			*		3	*						
4		*	*					4						*	*
5					*	*	*	5	*						
6		*	*			*	*	6	*					^a^	
7		*	*			*		7						*	
8					*	*		8					*	*	*
9		*	*			*		9	*						
10				*	*		*	10						*	
11		*	*		*		*	11	*						
12					*	*	*	12						*	*
13				*	*	*		13						*	
14		*				*	*	14						*	
15					*	*		15						*	
16				*	*	*		16	*						
–								17	*						
–								18						*	*
Total	–	9/16	8/16	3/16	9/16	13/16	7/16		7/18	–	–	–	1/18	11/18	4/18

Abbreviations: AI, abnormal imagina; DA, acute dystonia; DI, insidious dystonia; MC, metabolic crisis; MD, motor delay; MH, macrocephaly; N, no features of GA1.

^a^ No imaging done at diagnosis.

* indicates a translation stop codon.

### 3.3 Clinical outcomes

Of the three patients with LE GA1, one presented clinically in crisis that was fatal at 4 years of age, while that patient's younger sibling subsequently diagnosed by HRS did well. The third patient with low‐excretor phenotype was diagnosed aged 5 years by genome sequencing having presented with motor delay and chronic dystonia. MRI showed putamen hyperintensity which is suggestive for insidious onset dystonia. The patient's C5DC was normal and initial urine organic acid analysis was reported as normal but upon re‐analysis the patient was found to have elevated 3(OH)‐glutarate. Fibroblast GCDH enzyme analysis found there was 20% residual GCDH enzyme activity which decreased at higher temperature.

Seven patients died by school‐age, two in the HRS group (Table 1). Of these two, one died as a neonate due to sepsis; the exact contribution of GA1 to the cause of death was not established, while the other died aged 4 years following a respiratory infection and failure to implement the emergency protocol quickly. Two additional patients from the clinically presenting group died after 7 years of age. One patient is lost to follow‐up in adulthood and the remainder are alive. One patient in the HRS group was 4 years old and not yet in school but had no motor or learning disability. The first patient to be diagnosed by NBS was a 14 month‐old male and he continues to be asymptomatic. His sister was diagnosed by HRS at 13 months of age and at age 3 years she has mild speech delay, macrocephaly and abnormal MRI brain but normal motor development. Patients diagnosed clinically who survived to school‐age were more likely to have significant cerebral palsy and dystonia by school‐age (7/11; 63.6% vs. HRS and NBS 0/13; 0%, *p* = 0.001; chi‐squared, Table 1). Patients who presented clinically were also less likely to be in mainstream school versus HRS patients (5/10; 50% vs. 12/13; 92.3%, *p* = 0.012 chi‐squared). Incidence of epilepsy was similar in both groups (5/16; 31.3% in the clinical group and 3/18; 16.7% in the HRS group, *p* = 0.317; chi‐squared). All patients had generalised tonic clonic epilepsy, bar one patient diagnosed by HRS who had juvenile myoclonic epilepsy (see below).

### 3.4 Clinical events in older children

**FIGURE 1:**
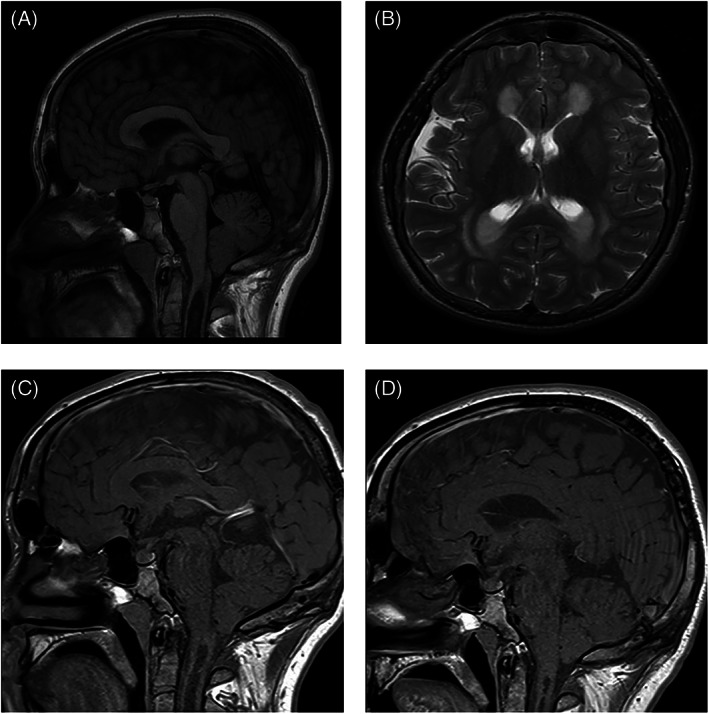
Sagittal T1 MRI of male patient diagnosed by HRS as a neonate aged 16 demonstrates mild inferior herniation of the cerebellar tonsils with a syrinx of the cervical cord (A) and hyperintensity of the white matter and corpus striatum on Axial T2 MRI in keeping with GA type I (B). Follow‐up midline sagittal T1 images shows interval improvement of the cervical cord syrinx aged 22 (C) and 27 (D) years.

A number of clinical events were observed in our patient population after 6 years of age, the traditional ceiling above which it is thought serious neurological abnormality does not occur.^5^ Three male patients who are homozygous for c.1093G>A, developed unusual clinical phenomena. One male patient, diagnosed in infancy by HRS developed spastic diplegia, with onset of symptoms at 8 years. This deterioration occurred following periods of erratic synthetic protein intake, which stabilised at 12 years of age. MRI brain and spine suggested no direct anatomical cause, EMG was equivocal and muscle biopsy suggested neurogenic atrophy. Neuroimaging performed showed typical features of GA1 (T2 hyperintensity of the most medial aspects of the lentiform nuclei bilaterally, mild–moderate ventricular dilatation, atrophy at the sylvian fissure, subdural collections at both temporal horns, arachnoid cysts) while on his most recent MRI aged 21, there was a finding of ventricular wall nodules. The patient was seen by a specialist in neuromuscular disease and no other contributory diagnosis made, symptoms stabilised after improved adherence to diet. The second male patient, diagnosed in the neonatal period having presented symptomatically with hypotonia, jitteriness, poor feeding and intermittent unexplained hyperpyrexia, developed new onset epilepsy unrelated to metabolic decompensation aged 16, after a number of stable years on treatment. These were generalised tonic–clonic seizures that were controlled with levetiracetam; of note there was also a family history of epilepsy unrelated to GA1. In the same time‐frame, this patient experienced pubertal delay and related reduction in height velocity aged 14 with no pituitary pubertal expansion on MRI aged 16. The third patient was diagnosed by HRS as a neonate and had a stable treatment course in early childhood, with concerns regarding adherence to prescribed treatment from 6 years onwards. He had MRI findings typical of GA1 in childhood without changes in the basal ganglia. Reduced height velocity beginning aged 11 years prompted investigation leading to a diagnosis of growth hormone deficiency and hypothyroidism. An MRI scan aged 16 revealed the presence of a cervical syrinx and Chiari I malformation (Figure 1A). Thickening of the pituitary stalk was also noted. There was no increase in admissions or metabolic crises in the years before the manifestation of central endocrine disorders or structural brain abnormalities. He received no neurosurgical intervention and follow‐up scans aged 22 and 27 showed interval improvement (Figure 1C,D).

**FIGURE 2:**
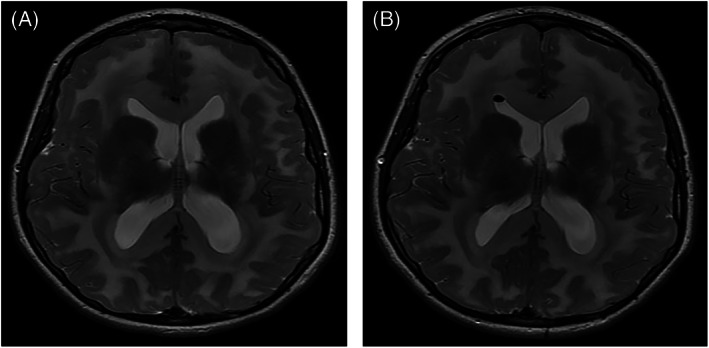
Axial T2 MRI of female patient diagnosed by HRS, aged 14 years demonstrates mild ventricular dilatation. Ventricles are larger than on baseline prior MRI studies. There is extensive hyperintensity of the white matter in keeping with GA type I (A). Axial T2 MRI obtained 1 week later (B) and 1 day post an Endoscopic Third Ventriculostomy (ETV) shows an interval slight decrease in ventricular size. There is a small bubble of air in the nondependent aspect of the right lateral ventricle.

Left thalamic haemorrhagic stroke was noted in a female patient aged 6 years with severe pre‐existing motor disability and on carnitine supplement only due to carer preference. The genotype of this patient is not known. Another female patient, unrelated to those mentioned above and with genotype c.344G>A;c.743C>T, developed juvenile myoclonic epilepsy and symptomatic ventriculomegaly with Chiari I malformation (Figure 2), aged 11 and 14 years, respectively. This patient had been diagnosed in infancy by HRS and there were no concerns regarding treatment adherence prior to development of these complications; she also had a family history of epilepsy. Ventriculomegaly was treated with endoscopic third ventriculostomy.

## 4 DISCUSSION

Here we report the outcomes of the Irish cohort of patients with GA1 with a cumulative follow‐up of 438 patient years. Over the 37 years of caring for patients with GA1 described, treatment practices have evolved, making some comparisons between outcomes over time difficult. Furthermore, this being a retrospective study, patient data were sometimes incomplete and this has led to missing information for certain data points.

We continue to show the positive effect early diagnosis and treatment, through ‘high‐risk’ screening, has on reduction of manifestations of GA1 such as reduction in MD at school‐age and improved attendance at mainstream school. We acknowledge the use of this as a proxy outcome, as formal cognitive testing was not carried out universally, however, in Ireland, it is more common practice for children with physical disability to attend mainstream school with supports, than for them to attend a special school. The rate of epilepsy was similar in both groups, which may in part be explained by other independent factors such as family history, or a general higher incidence of neurological disease within the Irish Travelling Community, the ethnic minority group to which 58% of Irish patients with GA1 belong. As presented in this study, unusual clinical events occurred in Irish patients aged over 6 years. The aetiology of these events is uncertain and to the authors' knowledge some have not previously been described in patients with GA1 either on or off treatment. Regarding pathophysiological mechanisms for the events described, we feel there is potential for a contributory role of GA1. For example, regarding the case of spastic diplegia, the only pathological finding was neurogenic atrophy on muscle biopsy, which could be explained by neurotoxic effect of the disease. Furthermore symptoms stabilised after improved adherence to diet. The acquired abnormalities of spinal syrinx and acquired Chiari I formation are outside of the typical neurosurgical abnormality of subdural effusions seen in GA1. Acquired Chiari 1 malformation is rare and is usually caused by a CNS tumour.^26^ We postulate that Chiari I malformations could develop due to worsening cerebral atrophy and accompanying increases in extra‐axial space and fluid, in the context of GA1. We also report central endocrine abnormalities; growth hormone and thyroid deficiency which are not frequently reported in patients with GA1 or other organic acidurias.^27^ Again we hypothesise these abnormalities could arise from parenchymal injury as a result of neurotoxic substances. Regarding the case of thalamic stroke, the combined role of vascular and metabolic components to stroke in GA1 has previously been explored.^28^ We note that three male patients with GA1 who share the pathogenic variant c.1093G>A experienced late‐onset neurological symptoms, could indicate a degree of genotype correlation with *clinical* phenotype, previously not seen in GA1.^29^ All patients who developed late complications had a HE biochemical phenotype.

While *insidious* type GA1 is described in patients diagnosed by NBS who have poor adherence to diet,^7^, ^16^ some of the clinical events reported here appear similar those described in patients with ‘*late‐diagnosis*’ GA1,^9^, ^18^, ^19^, ^20^, ^21^, ^22^, ^23^, ^24^ albeit in patients already on dietary intervention. However, in some of the examples reported new symptoms arose after periods of reduced adherence to dietary regimes and/or erratic protein intake. The authors feel these cases support the need for further research as to whether dietary therapy should continue lifelong.

GA1 is a rare disease, the adverse outcomes for patients can be devastating and new symptoms can occur remote in time from alterations in treatment, meaning traditional research approaches used for common diseases to guide therapeutic decisions are difficult to apply. Further research is needed as to whether late onset complications could be prevented by lifelong adherence to dietary protocols or until such time as new treatment modalities such as enzyme or molecular therapeutic approaches become available.^30^

## AUTHOR CONTRIBUTIONS

Lydia Healy contributed to study design, collection, analysis and interpretation of data, and co‐wrote the manuscript; Meabh O'Shea contributed to collection and interpretation of data, and co‐wrote the manuscript; Jenny McNulty provided guidance on dietary management of patients and contributed to editing the final manuscript; Graham King contributed to collection of data and edited the manuscript; Eilish Twomey reviewed and selected radiological images, wrote the legends and contributed to editing the final manuscript; Ellen Crushell provided clinical data and contributed to editing of the final manuscript; Joanne Hughes provided clinical data and contributed to editing of the final manuscript; Eileen Treacy provided clinical data; Ina Knerr provided clinical data, and contributed to the interpretation of data and editing of the manuscript; Ahmad Ardeshir Monavari provided clinical data, designed and supervised the study, co‐analysed the data and co‐wrote the manuscript. All authors were involved in managing the patients and/or collecting data over the years. All authors reviewed the final manuscript before publication.

## FUNDING INFORMATION

This research did not receive any specific grant from funding agencies in the public, commercial, or not‐for‐profit sectors.

## CONFLICT OF INTEREST

The authors declare no conflict of interest.

## ETHICS STATEMENT

This study was approved by the Research and Ethics Committee at Children's Health Ireland at Temple Street Hospital with the study number 19.042 and informed consent was obtained as appropriate.

## Data Availability

Due to the nature of this research, participants of this study did not agree for their data to be shared publicly, so supporting data are not available.
